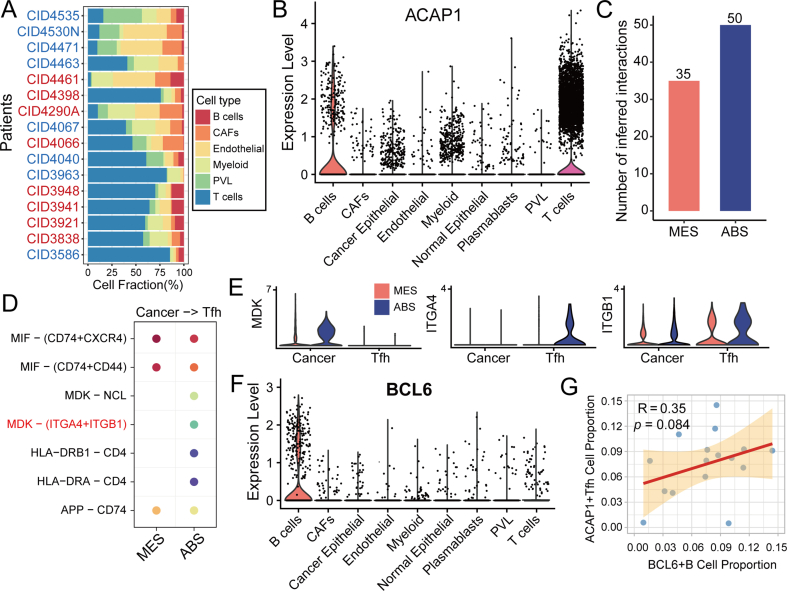# Alternative splicing of immune-related genes identifies breast cancer subtypes with differential immune cell infiltration

**DOI:** 10.1016/j.gendis.2024.101349

**Published:** 2024-06-14

**Authors:** Zhangxiang Zhao, Yuquan Wang, Zixin Jin, Huiming Han, Bo Chen, Mingyue Liu, Kaidong Liu, Shuping Zhuang, Haihai Liang, Yunyan Gu

**Affiliations:** a The Sino-Russian Medical Research Center of Jinan University, The Institute of Chronic Disease of Jinan University, The First Affiliated Hospital of Jinan University, Guangzhou, Guangdong 510632, China; b Department of Pharmacology (State-Province Key Laboratories of Biomedicine-Pharmaceutics of China, Key Laboratory of Cardiovascular Research, Ministry of Education), College of Pharmacy, Harbin Medical University, Harbin, Heilongjiang 150081, China; c Department of Systems Biology, College of Bioinformatics Science and Technology, Harbin Medical University, Harbin, Heilongjiang 150081, China

Breast cancer, recognized as a foremost cause of mortality among women, is characterized by a complex immune microenvironment. Alternative splicing (AS) is a crucial process leading to diverse transcript variants. Studies have shown AS's role in T cell and B cell stimulation. For instance, Shalek et al performed single-cell RNA sequencing and showed that different AS patterns existed depending on the maturity and differentiation of dendritic cells, such as Cxcl10.^1^ AS of *CD28* enhances the viability of activated T cells.^2^ However, the role of AS in breast cancer, particularly in relation to immune cell infiltration, remains underexplored.

We found 416 AS events in 782 immune-related genes out of a total of 30,846 AS events in 9636 genes.^3^ In AS events of immune-related genes, six splicing patterns were significantly enriched (Fig. S1A). For patients with non-basal breast cancer, the AS-related subtypes maintained a difference in survival (Fig. 1A). We found 12,371 differentially expressed genes between AS-related subtypes (adjusted *P* < 0.05, Wilcoxon rank-sum test). Highly expressed genes showed enrichment in focal adhesion and spliceosome pathways, indicating a divergence in phenotypes related to epithelial-mesenchymal transition and RNA splicing processes (Fig. S1B). Spliceosomes and epithelial-mesenchymal transition hallmark scores were significantly different between the two subtypes (Fig. 1B). We named the two subtypes MES and ABS, respectively. Similarly, the prognosis of MES was worse than ABS in each PAM50 subtype (Fig. S1C–E). Furthermore, classic mesenchymal marker genes and immune inhibitor *CD274* (PD-L1) showed higher expression in the MES subtype than the ABS subtype (Fig. S1F). In addition, patients with the MES subtype had a higher percentage of lymph node metastases and distant metastases (Fig. S1G).

**Figure 1:**
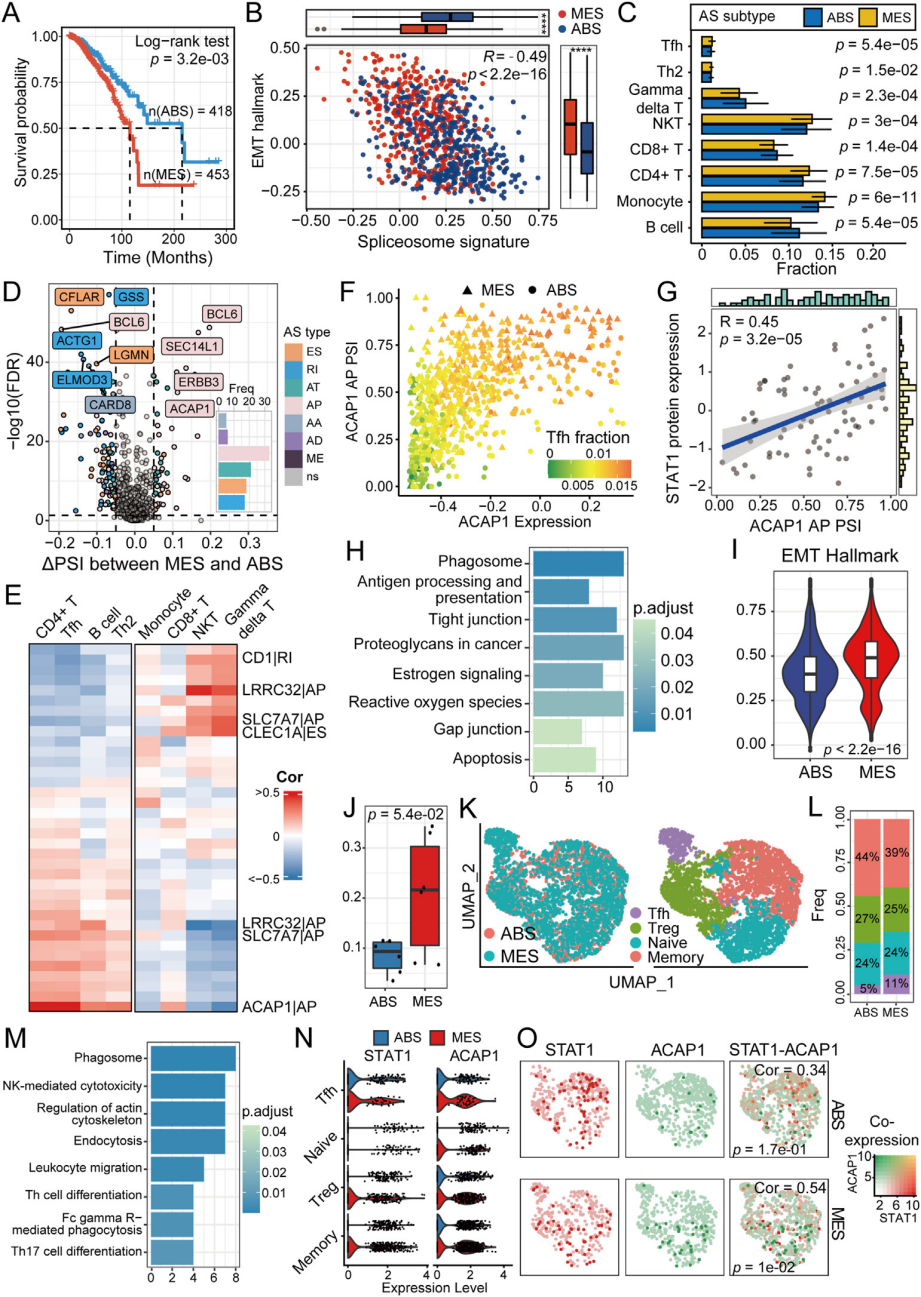
Alternative splicing (AS) of immune genes revealed *STAT1*-regulated alternative promoter of *ACAP1* in different breast cancer subtypes. **(A)** Kaplan–Meier plot for overall survival between MES and ABS subtypes. **(B)** Gene set scores of epithelial-mesenchymal transitions (EMT) by ssGSEA. **(C)** Infiltration of various immune cells of AS-related subtypes. **(D)** Differential spliced events between MES and ABS. **(E)** Pearson's correlation between infiltration of immune cells and differential spliced events. **(F)** Scatter plot of alternative promoter (AP) event and expression level of *ACAP1*. Dots were colored by the Tfh fraction. **(G)** Pearson's correlation between *STAT1* protein expression and percentage spliced-in (PSI) of *ACAP1* AP event. **(H)** Functional enrichment of differentially expressed genes between MES and ABS cancer cells. **(I)** EMT scores of MES and ABS cancer cells. **(J)** Fraction of *CD4*^*+*^ T cells in MES and ABS subtype. **(K)** UMAP visualization of 3855 *CD4*^*+*^ T cells colored by AS-related subtype and cell subset. **(L)** Fraction of *CD4*^*+*^ T cell subsets in MES and ABS subtype. **(M)** Functional enrichment of genes that correlated with *ACAP1* expression. **(N)***STAT1* and *ACAP1* expression in T cell subsets, split by AS-related subtypes. **(O)** Co-expression of *STAT1* and *ACAP1* in MES and ABS Tfh cells.

Subsequently, we found differences in tumor-microenvironment scores between ABS and MES subtypes (Fig. S1H). The ABS subtype exhibited higher infiltration levels of gamma delta T cells, CD8^+^ T cells, and B cells, whereas the MES subtype showed enrichment in monocytes, CD4^+^ T cells, and T follicular helper (Tfh) cells (Fig. 1C). The higher divergence in T cell receptor repertoires, as indicated by Shannon and Richness scores, supported a potential activation of CD4^+^ T cells in the MES subtype (Fig. S1I).

To assess the robustness of AS-related subtypes, we used 39 prognostic and differentially spliced genes to build a LASSO regression model (Fig. S2A). MES samples from METABRIC and GSE96058 datasets consistently showed worse prognosis than ABS samples (Fig. S2B, C). A similar tendency was observed in two datasets detected by microarray, GSE21653 and GSE10886 (Fig. S2D, E). Regarding tumor-infiltrating immune cells, MES samples demonstrated higher levels of infiltration by activated dendritic cells, Tfh cells, and memory CD4^+^ T cells than ABS subtype in both METABRIC and GSE96058 (Fig. S2G, H).

To assess the relationships between AS events and immune cell infiltration, we identified a total of 16,825 differential AS events across 7072 genes between the MES and ABS subtypes, including splicing events of *BCL6*, *ERBB3*, and *SEC14L1* (Fig. 1D). The infiltration levels of Tfh cells and CD4^+^ T cells were significantly correlated with the usage of promoters upstream of the first exon of *ACAP1* (Fig. 1E). Furthermore, the alternative promoter event of *ACAP1* was positively correlated with *ACAP1* expression (Fig. 1F). Similarly, both the expression and percentage spliced-in level of *ACAP1* were significantly and positively correlated with the fraction of Tfh cells.

To identify regulatory factors influencing the alternative promoter event of *ACAP1*, we investigated common differentially expressed genes across the TCGA, METABRIC, and GSE96058, identifying 77 RNA binding proteins and 218 transcript factors (Fig. S3A). Among these regulatory factors, three RNA binding proteins and four transcript factors were consistently differentially expressed at the proteomic level, including up-regulated *STAT1* and *NFKB2* in MES samples (Fig. S3B). The protein and RNA expression of *STAT1* were highly positively correlated (Fig. S3C). Meanwhile, *STAT1* was the protein most correlated to percentage spliced-in values of *ACAP1*, indicating that *STAT1* is potentially a major transcript factor regulating the alternative promoter event of *ACAP1* (Fig. 1G). Furthermore, amino acid quantification by mass spectrometry supported an elevated expression of the *ACAP1* protein in the MES subtype (Fig. S3D). We also found that upstream 2 kb and 40 kb regions of *ACAP1* had higher accessibility in MES samples (Fig. S3E). Furthermore, we discovered that five predicted *STAT1* binding sites were located 3 kb upstream in the open region from the first exon of *ACAP1*, as determined by TCGA ATAC-sequencing data and the JASPAR database (Table S1).^4^

We then confirmed the correlation between *STAT1* and *ACAP1* expression in Tfh cells at the single-cell level. Using matched bulk and single-cell RNA-sequencing samples from GSE176078 and the LASSO regression, samples were classified as MES and ABS subtypes (Fig. S4A). Differentially expressed genes between MES and ABS cancer cells were enriched in estrogen receptor signaling pathways and cell junction functions (Fig. 1H). Moreover, MES cancer cells showed a higher epithelial-mesenchymal transition hallmark score than ABS cancer cells (Fig. 1I).

In non-tumor cell types, only the fraction of CD4^+^ T cells showed a marginally significant difference (Fig. 1J). We clustered CD4^+^ T cells and found four main subsets including memory T cells, *FOXP3*^+^ regulatory T cells, *CXCL13*^+^ Tfh cells, and naïve cells (Fig. 1K). Memory T cells and regulatory T cells constituted a higher proportion in ABS samples, while MES samples had more Tfh cells (Fig. 1L). *ACAP1* was expressed highly in T cells and B cells (Fig. S4B). Gene co-expression networks associated with *ACAP1* expression of CD4^+^ T cells were related to T cell differentiation and cell adhesion function (Fig. 1M). Although *ACAP1* was expressed universally in all CD4^+^ T cell subsets from ABS and MES samples, *STAT1* was specifically highly expressed in Tfh cells compared with other CD4^+^ T cell subsets from MES samples (Fig. 1N). Furthermore, we found that the *ACAP1* and *STAT1* expression were significantly correlated in Tfh cells from MES samples, but not in those from ABS samples (Fig. 1O).

In terms of cell–cell communication, the Cellchat identified more inferred interactions in the ABS subtype than in the MES subtype (Fig. S4C). Within *MDK* signaling, *MDK*-*NCL* and *MDK*-*ITGB1* interactions were predicted as predominant between ABS cancer cells and Tfh cells (Fig. S4D). Our single-cell analysis indicated coexistence between Tfh cell infiltration and the expression of mesenchymal phenotypic markers of MES cancer cells. However, the MES cancer cell lowly expressed *MDK* gene, which interacts with Tfh cells by *ITGB1* (Fig. S4E). It is well-known that *ACAP1* promotes the accumulation of *ITGB1* in endosomes.^5^ Here, we proposed that MES cancer cells regulate the Tfh cell infiltration through the interaction between *ITGB1* and *MDK*. We found low expression of *BCL6* in T cells, indicating an alternative way for Tfh differentiation in breast cancer (Fig. S4F). Analyzing the potential interaction between *ACAP1* and *BCL6* expression revealed a significant correlation between *ACAP1*^+^ Tfh and *BCL6*^+^ B cells in the MES subtype (Fig. S4G).

In summary, our analysis identified two subtypes for non-basal breast cancer with a distinct prognosis based on AS events. Given that *ACAP1* is known to interact with *ITGB1*, the downstream signaling pathways triggered by the *ACAP1*-*ITGB1* interaction merit further experimental investigation. Additionally, our findings underscore the value of conducting *in vivo* studies to examine the therapeutic possibilities of targeting the *STAT1*-*ACAP1* axis to modulate Tfh cell differentiation or migration in breast cancer. This study highlights the significance of AS events in immune-related genes for the infiltration and differentiation of immune cells in breast cancer.

## Ethics declaration

Informed consent was obtained from all subjects involved in the study.

## Author contributions

Conceptualization, Z.X.Z., Y.Q.W., H.H.L., and Y.Y.G.; methodology, Z.X.Z. and Y.Q.W.; software, Z.X.Z., Y.Q.W., B.C., and H.M.H.; formal analysis, Z.X.Z., Y.Q.W., and B.C.; writing – original draft, Z.X.Z. and Y.Q.W.; writing – review & editing, Z.X.Z., Y.Q.W., M.Y.L., K.D.L., H.H.L., and Y.Y.G.; funding acquisition, Z.X.Z., H.H.L., and Y.Y.G.; resources, S.P.Z. and Z.X.J.; supervision, H.H.L. and Y.Y.G. All authors read and approved the final manuscript for submission.

## Conflict of interests

The authors declared no conflict of interests.

## Funding

This work was supported by the National Key Research and Development Program of China (No. 2023YFF1204600), the Guangdong Province Basic and Applied Basic Research Fund (China) (No. 2022A1515110765), the National Natural Science Foundation of China (No. 32270710), and the Outstanding Youth Foundation of Heilongjiang Province of China (No. YQ2021H005).

## Figures and Tables

**figs1:**
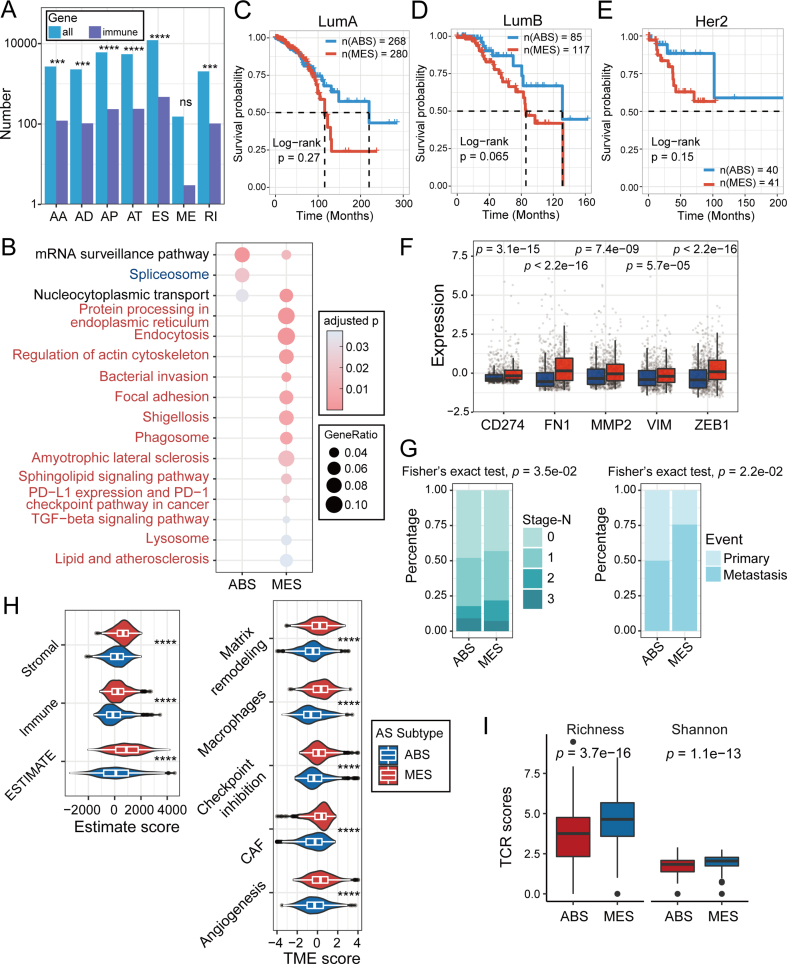


**figs2:**
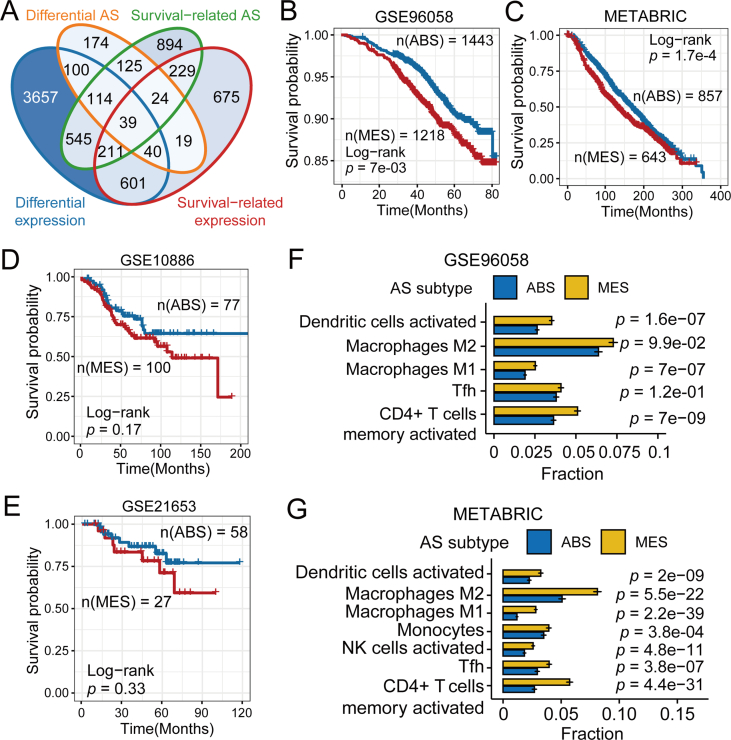


**figs3:**
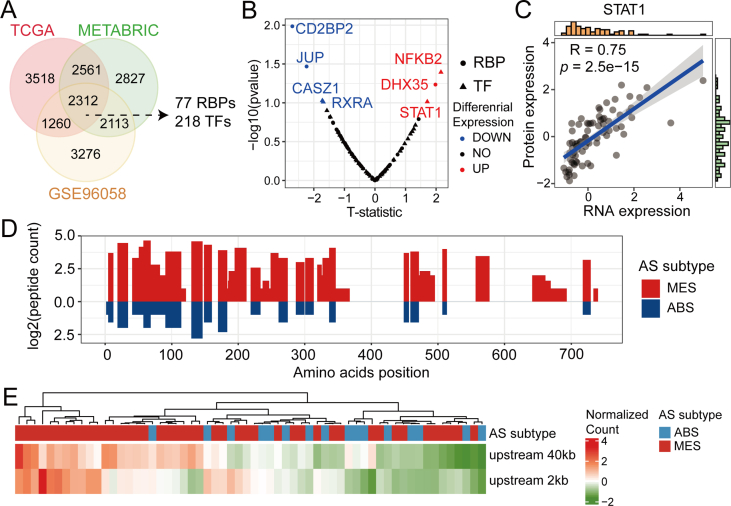


**figs4:**