# Supplementary material for: Alternative splicing of immune-related genes identifies breast cancer subtypes with differential immune cell infiltration
Source: Genes Dis. 2024 Jun 14;12(2):101349. doi: 10.1016/j.gendis.2024.101349 (PMC11625315; doi:10.1016/j.gendis.2024.101349)
Supplement: Multimedia component 1 [file mmc1.docx]

**Supplementary Materials**

Contents

[**Figure S1 2**](#_Toc155789426)

[**Figure S2 4**](#_Toc155789427)

[**Figure S3 5**](#_Toc155789428)

[**Figure S4 6**](#_Toc155789429)

[**Methods and Material 7**](#_Toc155789430)

[**References 10**](#_Toc155789431)

# Figure S1


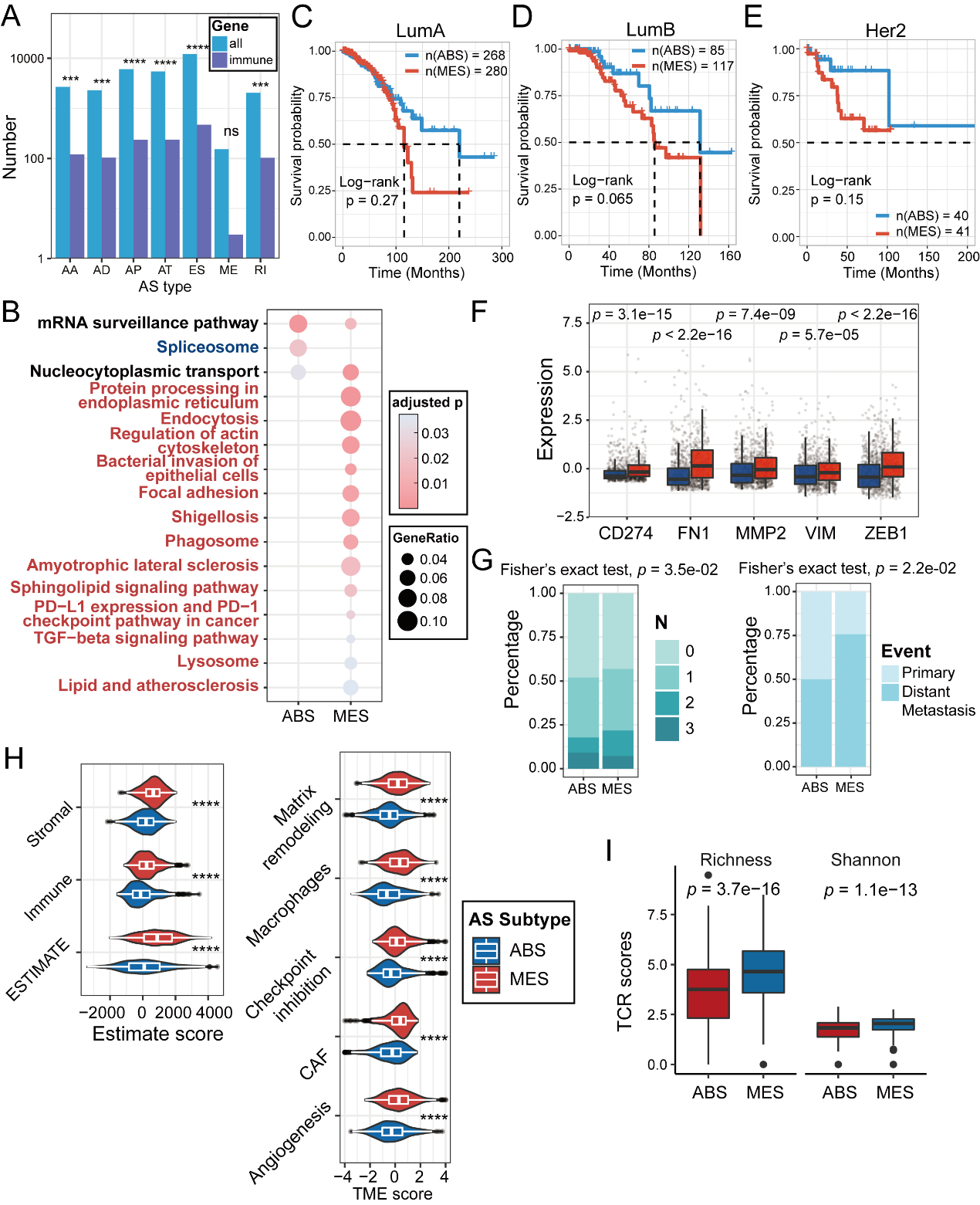


**The independence of survival and functional differences between AS-related subtypes stratified by PAM50 subtypes.** (**A**) Number of AS events in immune-related genes versus all genes, ordered by AS type and tested by Fisher’s exact test (SE, skipped exon; AP/AT/AD/AA, alternative promoter/terminator/donor/acceptor; RI, retained intron). (**B**) Interaction size and enrichment significance of up-regulated genes for AS-related subtypes. (**C-E**) Kaplan–Meier plot for overall survival in each PAM50 subtype. (**F**) Differential expressed mesenchymal markers and CD274. (**G**) Percentages of patients with lymph node or distant metastases. (**H**) Differential immune and stromal score by ESTIMATE, and tumor microenvironment scores. (**I**) TCR scores of AS-related subtypes. *, P < 0.05; **, P < 0.01; ***, P < 0.001; ****, P < 0.0001; ns, non-significance.

# Figure S2


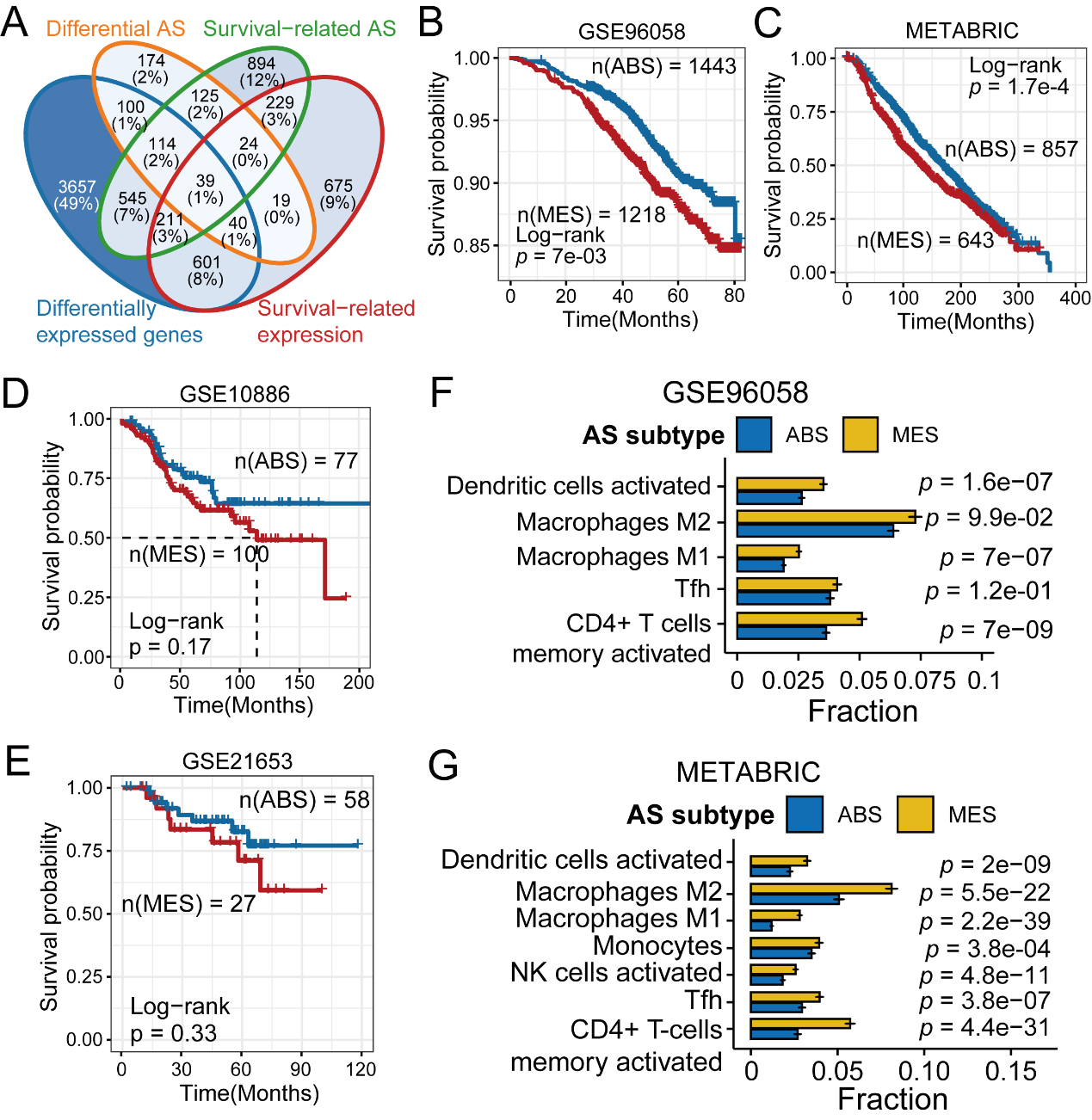


**Survival analysis and immune infiltration of AS-related subtypes in independent datasets.** (**A**) Interaction among differentially expressed genes, survival-related genes, differential spliced events, and survival-related events. (**B-E**) Kaplan–Meier plot for overall survival of MES and ABS in (**B**) GSE96058, (**C**) METABRIC, (**D**) GSE10886 and (**E**) GSE21653. (**F-G**) Differential infiltration of immune cells in (**F**) GSE96058 and (**G**) METABRIC.

# Figure S3


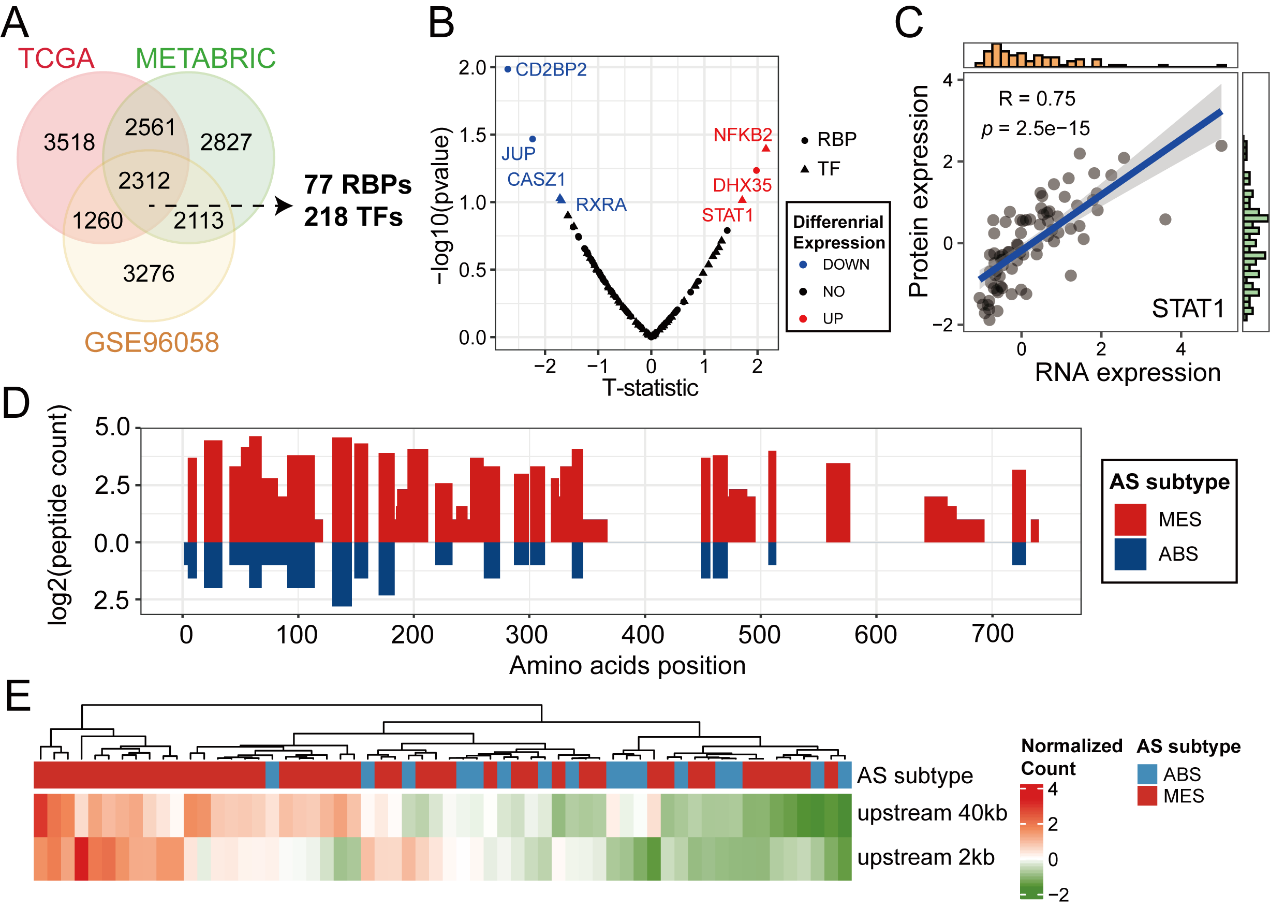


**Correlation between the *ACAP1* alternative promoter (AP) event and STAT1 protein.** (**A**) Interaction of differential expressed transcription factors (TFs) and RNA-binding proteins (RBPs) among TCGA, METABRIC, and GSE96058. (**B**) Volcano plot shows differentially expressed TFs and RBPs at proteomic level. (**C**) Pearson’s correlation between *STAT1* RNA and protein expression. (**D**) Overall counts of detected peptides along with the ACAP1 protein isoform. (**E**) Normalized count of two differential accessible regions by ATAC-seq. Samples were colored by AS-related subtype.

# Figure S4


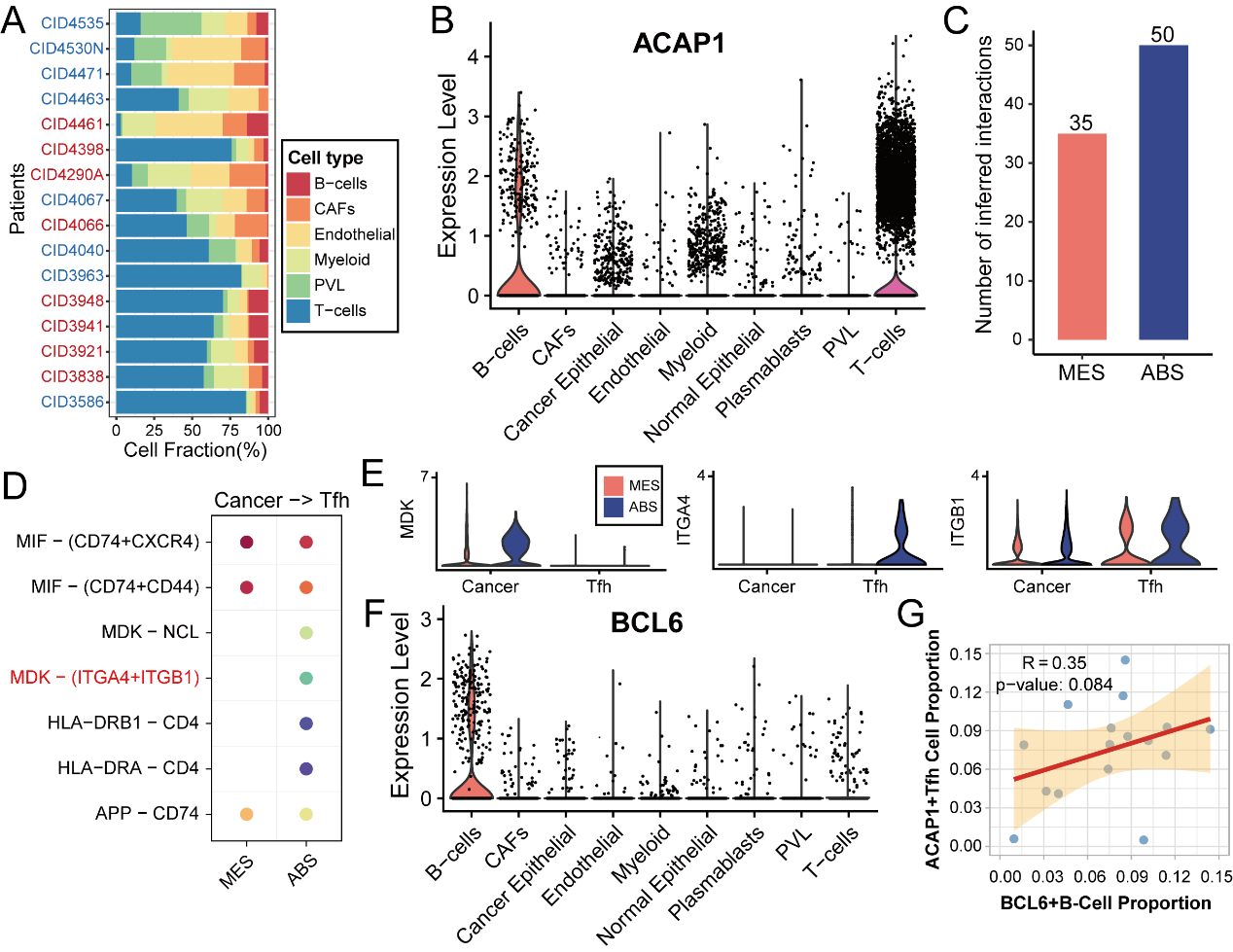


**Single-cell co-expression between the *ACAP1* expression and *STAT1* in T-cell follicular helper (Tfh) cells.** (**A**) The cell fraction of non-basal breast cancer samples, and samples were colored by AS-related subtype. (**B**) *ACAP1* expression in various cell types at single-cell level. (**C**) Number of inferred overall interactions by Cellchat among cancer cells and Tfh cells. (**D**) Ligand-receptor of differential signaling pathways. Nodes were colored by inferred probabilities. (**E**) The expression levels of *MDK*, *ITGA4*, and *ITGB1* in cancer and Tfh cells. (**F**) *BCL6* expression at the single-cell level. (**G**) The correlation between ACAP1+Tfh cell proportion and *BCL6*+B-cell proportion.

# Methods and Material

**Data acquisition and preprocessing**

The RNA-Seq data, copy number variation, DNA methylation, and clinical information of TCGA-BRCA patients and METABRIC were obtained from cBioPortal (http://www.cbioportal.org/). Proteomic mzid files of TCGA were downloaded from CPTAC (http://proteomics.cancer.gov/). Gene expression profiles of GSE96058, GSE10886, GSE21653, and GSE176078 were downloaded from GEO database (https://www.ncbi.nlm.nih.gov/geo/). All basal breast cancer samples were removed.

mRNA SpliceSeq data was downloaded from the TCGA SpliceSeq (https://bioinformatics.mdanderson.org/TCGASpliceSeq/). To obtain more reliable AS events, a series of strict filters (percentage of Samples with PSI value ≥75, average of PSI value ≥0.05) were implemented resulting in the identification of 35,367 AS events from 10,274 genes.

Table S2 shows the statistics of samples in this study.

Gene signatures of 28 tumor-infiltrating immune cells were downloaded from TISIDB (http://cis.hku.hk/TISIDB/download.php). The DNase-seq signal of multiple biosamples was queried in the SCREEN database (https://screen.encodeproject.org/)^1^. Predicted STAT1 binding sites were downloaded from JASPAR database (https://jaspar.genereg.net/)^2^.

**The lasso regression model for AS subtype**

Candidate feature genes were defined as prognosis differentially expressed and spliced genes by Cox univariant regression and Wilcoxon rank-sum test (adjusted *P* < 0.05). TCGA non-basal samples were randomly split into a training and test dataset in a 9:1 ratio. Parameters were tunning using 20-fold cross-validation and the loss function was defined as mean squared error. Regression was performed by glmnet function using parameters with the minimum error^3^.

**Estimation of TME scores and fractions of immune cells**

Tumor purity, stromal score, immune score and TME scores were generated using ESTIMATE software by the TGCA research^4^. The relative fraction of immune cells for TCGA, METABRIC, and GSE96058 was estimated using CIBERSORT and ImmuneCellAI software^5, 6^.

**Mass spectrometry-based quantitative method**

Proteomic mzid files from CPTAC database were imported by mzID R package. For each proteomic peptide-level profile, we adjusted the log-transformed expression values to calculate standard deviations away from the median. Subsequently, we standardized the expression values across different samples, again calculating standard deviations from the median. In the peptide spectral matches from CPTAC, the ACAP1 peptides were obtained using UNIPROT accession Q15027. For visualization, the peptide positions were mapped to gene body.

**Functional enrichment analysis**

Gene set enrichment analysis were performed by clusterProfiler package with KEGG and GO annotation^7^. The ssGSEA algorithm of GSVA package was used to score MsigDB hallmark gene sets (https://www.gsea-msigdb.org/gsea/msigdb/)^8, 9^.

**Single cell analysis**

We utilized the Seurat R package to perform single cell analysis on single cell profile from GSE176078^10^. We retained genes that were expressed in more than four cells and cells that contained at least 200 genes. Cells with more than three median absolute deviations away from median number of genes, median percentage of mitochondrial or ribosome genes were removed.

For CD4+ T-cell clustering, data matrix was transformed into log-space and ‘vst’ selection was performed to identify top 1,000 highly variable genes. These highly variable genes were used to perform PCA and top five principal components to calculate shared nearest neighbors. After batch correction by the Harmony package, cells were clustered by original Louvain algorithm (resolution = 0.2)^11^. Using Seurat function ‘FindMarker’, DEGs between two clusters were identified with minimum percentage > 0.2 and log2(fold change) > 1. The pseudo-time adjacent analysis was performed by slingshot package with the default threshold^12^.

We used CellChat (v1.0.0) to evaluated cell-cell interactions and significant pathways^13^. Normalized data were loaded into CellChat. We used ‘CellChatDB_human’ as the ligand-receptor dataset. Default parameters were used to identify putative interaction pairs with more than 10 cells in each cell group.

**Statistical analysis and visualization**

All statistical analysis was performed and visualized based on R (v4.1.1). For quantitative data, Student’s two-sided T test and Wilcoxon’s rank-sum test were used to estimate statistical significance for normally distributed variables and non-normally distributed variables, respectively. The spearman’s rank correlation was used to measures the strength and direction of association between two variables. P-values were adjusted by the Benjamini-Hochberg Procedure.

# References

1. Consortium EP, Moore JE, Purcaro MJ, Pratt HE, Epstein CB, Shoresh N, Adrian J, Kawli T, Davis CA, Dobin A, et al. Expanded encyclopaedias of DNA elements in the human and mouse genomes. Nature 2020; 583:699-710.

2. Castro-Mondragon JA, Riudavets-Puig R, Rauluseviciute I, Lemma RB, Turchi L, Blanc-Mathieu R, Lucas J, Boddie P, Khan A, Manosalva Perez N, et al. JASPAR 2022: the 9th release of the open-access database of transcription factor binding profiles. Nucleic Acids Res 2022; 50:D165-D73.

3. Friedman J, Hastie T, Tibshirani R. Regularization Paths for Generalized Linear Models via Coordinate Descent. J Stat Softw 2010; 33:1-22.

4. Bagaev A, Kotlov N, Nomie K, Svekolkin V, Gafurov A, Isaeva O, Osokin N, Kozlov I, Frenkel F, Gancharova O, et al. Conserved pan-cancer microenvironment subtypes predict response to immunotherapy. Cancer Cell 2021; 39:845-65 e7.

5. Newman AM, Steen CB, Liu CL, Gentles AJ, Chaudhuri AA, Scherer F, Khodadoust MS, Esfahani MS, Luca BA, Steiner D, et al. Determining cell type abundance and expression from bulk tissues with digital cytometry. Nature Biotechnology 2019; 37:773-82.

6. Miao YR, Zhang Q, Lei Q, Luo M, Xie GY, Wang H, Guo AY. ImmuCellAI: A Unique Method for Comprehensive T-Cell Subsets Abundance Prediction and its Application in Cancer Immunotherapy. Adv Sci (Weinh) 2020; 7:1902880.

7. Yu G, Wang LG, Han Y, He QY. clusterProfiler: an R package for comparing biological themes among gene clusters. OMICS 2012; 16:284-7.

8. Hanzelmann S, Castelo R, Guinney J. GSVA: gene set variation analysis for microarray and RNA-seq data. BMC Bioinformatics 2013; 14:7.

9. Liberzon A, Birger C, Thorvaldsdottir H, Ghandi M, Mesirov JP, Tamayo P. The Molecular Signatures Database (MSigDB) hallmark gene set collection. Cell systems 2015; 1:417-25.

10. Butler A, Hoffman P, Smibert P, Papalexi E, Satija R. Integrating single-cell transcriptomic data across different conditions, technologies, and species. Nature Biotechnology 2018; 36:411-20.

11. Korsunsky I, Millard N, Fan J, Slowikowski K, Zhang F, Wei K, Baglaenko Y, Brenner M, Loh PR, Raychaudhuri S. Fast, sensitive and accurate integration of single-cell data with Harmony. Nature methods 2019; 16:1289-96.

12. Street K, Risso D, Fletcher RB, Das D, Ngai J, Yosef N, Purdom E, Dudoit S. Slingshot: cell lineage and pseudotime inference for single-cell transcriptomics. BMC genomics 2018; 19:477.

13. Jin S, Guerrero-Juarez CF, Zhang L, Chang I, Ramos R, Kuan CH, Myung P, Plikus MV, Nie Q. Inference and analysis of cell-cell communication using CellChat. Nature communications 2021; 12:1088.
